# Predicting change in symptoms and function in patients with persistent shoulder pain: a prognostic model development study

**DOI:** 10.1186/s12891-021-04612-y

**Published:** 2021-08-27

**Authors:** Mathias Moselund Rønnow, Thor André Brøndberg Stæhr, David Høyrup Christiansen

**Affiliations:** Department of Occupational Medicine, University Research Clinic, Regional Hospital West Jutland, Gl. Landevej 61, 7400 Herning, Denmark

**Keywords:** Prognosis, Prediction, Prognostic model, Exercise, Physical therapy, Physiotherapy, Shoulder pain, Chronic shoulder pain, Persistent shoulder pain, Shoulder disorders

## Abstract

**Background:**

Persistent shoulder pain causes considerable disruption of the individual’s life and imposes high costs on healthcare and society. Well-informed treatment and referral pathways are crucial as unsuccessful interventions and longer duration of symptoms minimizes the likelihood of success in future interventions. Although physiotherapy is generally recommended as first line treatment, no prognostic model or clinical prediction rules exists to help guide the treatment of patients with persistent shoulder pain undergoing physiotherapy.

Thus, the *objective* of this study was to develop a prognostic model to inform clinical decision making and predict change in symptoms and function in patients with persistent shoulder pain.

**Methods:**

This was a prospective cohort study of 243 patients with persistent shoulder pain referred to outpatient physiotherapy rehabilitation centres. Data was collected at baseline and six-month follow-up. The outcome was change in shoulder symptoms and function as measured by the shortened version of the Disabilities of the Arm, Shoulder and Hand questionnaire (QuickDASH) from baseline to 6 months follow up. Potential predictors were included in a multivariable linear regression model which was pruned using modified stepwise backwards elimination.

**Results:**

The final model consisted of seven predictors; baseline QuickDASH score, employment status, educational level, movement impairment classification, self-rated ability to cope with the pain, health-related quality of life and pain catastrophizing. Together these variables explained 33% of the variance in QuickDASH-change scores with a model root mean squared error of 17 points.

**Conclusion:**

The final prediction model explained 33% of the variance in QuickDASH change-scores at 6 months. The root mean squared error (model SD) was relatively large meaning that the prediction of individual change scores was quite imprecise. Thus, the clinical utility of the prediction model is limited in its current form. Further work needs be done in order to improve the performance and precision of the model before external validity can be examined along with the potential impact of the model in clinical practice. Two of the included predictors were novel and could be examined in future studies; movement impairment classification based on diagnosis and health-related quality of life.

**Supplementary Information:**

The online version contains supplementary material available at 10.1186/s12891-021-04612-y.

## Background

Shoulder pain is one of the most common musculoskeletal complaints in the general population and can mostly be managed in primary care [1, 2]. However, more than half of the people presenting with a new episode of shoulder pain develop persistent symptoms, which can lead to considerable disruption of the individual’s life [3, 4]. The patients who do not respond favourably to treatment in primary care are commonly referred to orthopaedic evaluation in secondary care [5, 6]. For common shoulder disorders, clinical guidelines recommend at least 3 months of physiotherapy with exercise before surgery is considered, which in Denmark frequently includes referral to the municipal outpatient rehabilitation centres [7]. As the patients have undergone longer care trajectories prior to the municipal rehabilitation (general practitioner – secondary care – municipal rehabilitation), they typically present with persistent symptoms and complications. Further unsuccessful treatment increases the likelihood of persistent symptoms and reduces the effectiveness of future interventions [8]. This can lead to both higher tangible and intangible costs (e.g. loss of quality of life), which is problematic for patients, clinicians, and for society [4, 9]. The economic impact of a relatively small amount of unsuccessfully rehabilitated patients was underlined in a study from Sweden, that estimated that one-fifth of shoulder patients were responsible for 91% of the total tangible costs [9]. To optimize the use of scarce resources and to improve the outcomes of these patients, it is necessary to gain a greater knowledge about which factors predict the prognosis of shoulder patients with persistent symptoms. A series of important prognostic factors can be combined in a prognostic model to predict future outcomes [10–14]. Based on the model, clinical prediction rules can be developed in order to tailor interventions to the individual patient and subgroups [10–14]. These can then be tested in intervention studies and can potentially lead to new and more effective treatment and referral pathways to the benefit of the patient and society. In recent years, several studies reporting on prognosis and prognostic models for shoulder patients undergoing physiotherapy have been published [15–23]. However, there is a lack of models with the potential to be used in clinical practice – especially in patients with long-term symptoms [15–17, 19–22].

Thus, the *aim* of this study was to develop a prognostic model to inform clinical decision making and predict change in symptoms and function in patients with persistent shoulder pain referred to outpatient physiotherapy rehabilitation.

## Methods

### Design and study population

This prospective cohort study was performed at six municipal outpatient rehabilitation centres in West Jutland, Denmark. From February 2018 to August 2019 consecutive patients referred for physiotherapy with shoulder pain were invited to participate. Inclusion criteria were: Age above 18 years, adequate understanding of the Danish language to complete questionnaires, and referral to physiotherapy as a part of conservative treatment. All patients received a confirmation letter on their rehabilitation referral along with written information about the project via their secure digital mailbox (e-Boks.dk) where Danish citizens receive all letters from public authorities [24, 25]. If agreeing to participate, the patients were asked to complete an online questionnaire before their first consultation. They were notified by email when follow-up questionnaires at two-, four- and six- months were available. Data collection was administrated by an online clinical database, Trial Partner (https://trialparner.clin.au.dk).

No attempts were made to control treatment, which was left up to the physiotherapist’s discretion. The study was approved by the Danish Data Protection Agency (no 1–16–02-9-18) and as referral and treatment pathways were unaffected by participation in the study, under Danish law, no ethics approval was needed (Act on Research Ethics Review of Health Research Projects, October 2018) [26].

Reporting of the present study follows the TRIPOD statement [27, 28].

### Outcome variable

The main outcome of interest was change in symptoms and function over 6 months (baseline score minus follow up score) as measured by the Danish version of the Quick Disabilities of the Arm, Shoulder and Hand questionnaire (QuickDASH). The QuickDASH is a patient reported outcome measure that measures physical function and symptoms of the upper extremity through 11 items. Scores are converted to a scale of zero to 100 where zero represents no symptoms and disability and 100 represents maximum symptoms and disability. The QuickDASH has been found to be acceptable, valid, reliable and responsive in various shoulder and upper-extremity patients [29–34].

### Candidate predictors

The selection of candidate predictors was based on previous literature and included sociodemographic, psychological and clinical characteristic variables (Table 1) [17, 19, 20, 35]. In addition to predictors from previous literature, three variables that had not been examined in shoulder patients before were included for modelling: Health-related quality of life, self-rated risk of persistent symptoms and movement impairment classification. We decided a priory to categorise diagnosis into movement impairments based on the work by Ludewig et al. since limited prognostic value has been found for most shoulder diagnoses (see Table 1) [36]. All candidate predictors were collected by a baseline questionnaire and registration forms from first consultation. Thus, the model was designed to predict expected change in symptoms and function over 6 months at first consultation.

**Table 1:** Candidate predictor overview

Predictor	Collection method/ source	Specification and/or *categories*
*Sociodemographics*		
Age	Unique identifier (CPR number)^a^	Years
Sex	Unique identifier (CPR number)^a^	*Male or female*
Professional educational level	Baseline questionnaire	*None, short-cycle higher education < 2½ years, medium-cycle higher education 3½-4 years, long-cycle higher education > 4 years, other*
Employment status	Baseline questionnaire	*Employed, subsidised employment, leave of absence, unemployed, student/ apprentice/ vocational training, early retiree/ retiree/ voluntary early retiree, other*
*Clinical characteristics*		
QuickDASH	Baseline questionnaire	As in “outcome variable”
Duration of symptoms	Baseline questionnaire	Months
Pain intensity	Baseline questionnaire; Numeric pain rating scale (NPRS) [37–39]	Typical shoulder pain the last 14 days ranging from 0 “no pain” to 10 “worst pain imaginable
Sick leave	Baseline questionnaire	Whole days with any sick leave due to current episode of shoulder pain
Movement impairment classification	Pathoanatomic diagnosis was assessed by the physiotherapist at first consultation and based on that, the patients were classified in movement impairment groups after data collection [36]	3 groups based on movement impairment: *Hypomobility (capsulitis, arthritis, post fracture* etc.*), Hypermobility (instability, trauma* etc.*), and Aberrant motion (rotator cuff, impingement, pain with movement* etc.*)*
*Pain behaviour and psychological factors*		
Fear avoidance	Baseline questionnaire; two questions from the Danish short form version of Örebro Musculoskeletal pain questionnaire [40–44]	Two questions ranging from 0 to 10 (0 = no fear avoidance, 10 = high fear avoidance) with a sum score from 0 to 20
Self-rated ability to cope with the pain	Baseline questionnaire; one questions from the Danish version of Örebro Musculoskeletal pain questionnaire [40–44]	One question ranging from 0 to 10 (0 = no ability, 10 = complete ability)
Self-rated risk of the pain becoming persistent	Baseline questionnaire; one question from the Danish version of Örebro Musculoskeletal pain questionnaire [40–44]	One question ranging from 0 to 10 (0 = no risk, 10 = very large risk)
Pain catastrophizing	Baseline questionnaire; two questions from the pain catastrophizing scale [45–48]	Two questions ranging from 0 to 10 with a sum score from 0 to 20 (0 = no pain catastrophizing, 20 = high pain catastrophizing)
Mental wellbeing	Baseline questionnaire; WHO Mental wellbeing Index (WHO-5) [49]	A five-item questionnaire with a sum score ranging from 0 (low mental wellbeing) to 100 (high mental wellbeing)
Health-related quality of life	EQ 5D-5L index [50, 51]	The index contains five dimensions each assessed by one question regarding; mobility, self-care, usual activities, pain/discomfort and anxiety/depression [52]. Utility values derived from a general population sample is used to calculate an index score ranging from − 0.6 to 1, where 1 represents a perfect health-related quality of life [52–54].

^a^CPR numbers contain information on both sex and age [55]

### Statistical analysis

#### Sample size

As a result of the multivariable nature of prognostic modelling studies, it is difficult to estimate the required sample size [11]. Thus, the number of variables allowed for inclusion was based on the assumption that at least 10 subjects are needed per degree of freedom in the model [11, 56].

#### Descriptive statistics and missing data

The baseline population was described by percentages and means, and by medians when numerical variables were not normally distributed. Number of missing values, follow-up response rate and differences in baseline characteristics between responders and non-responders were calculated. Candidate predictors with missing values > 10% were discarded since the problem is likely to recur when using the model in clinical practice [12]. Further, patients with missing values in included predictors were excluded.

#### Prognostic modelling

The prognostic modelling was performed by pruning a full multiple linear regression model which included all the candidate predictors using modified backwards elimination. Model performance was assessed by the adjusted coefficient of determination (adjusted R^2^), which summarizes the predictive ability of a normal-error model through its explained variance (in this case variance in QuickDASH-change) adjusting for number of included variables relative to the number of data points [14, 57, 58].

Pruning was done by:

1. Presuming overlap in predictor information based on the literature and clinical reasoning [28]. To get an indication of whether the presumption was fair, the correlation between variables was examined with Spearman’s correlation, since high correlations indicate that one of the variables does not add much to the prediction [12]. Variables were eliminated only if it did not lead to a drop in the adjusted R^2^.
2. When no more presumptions were present, correlations, standardized coefficients and *p*-values were used to eliminate the next variable [57]. Deletion of a variable could not lead to a higher drop in the adjusted R^2^ value than 0.5%.

Before pruning, two variables, employment status and professional educational level, were each collapsed into three categories to reduce the number of degrees of freedom of the full model. From diagnostic plots, both the full and the final model were deemed in line with the assumptions of multiple linear regression. Internal validation was performed by bootstrapping the final model (10.000 reps) to obtain a confidence interval for the adjusted R^2^.

A sensitivity analysis was performed to examine the model’s robustness to extreme QuickDASH change-scores in patients lost to follow up: The prognostic modelling was carried out on two scenarios: A best case scenario where patients with missing change-scores were assigned predicted 6 months change-scores plus the minimal important change (MIC) of 13.6 points, and a worst case scenario where the MIC was subtracted from the predicted change scores [31]. The performance of the final model was compared to the performance of a parsimonious model including only baseline QuickDASH as a predictor and a supplementary analysis was performed using Akaike’s information criteria instead of the adjusted coefficient of determination for the backwards elimination [59]. To allow for comparison across supplementary models we only included participants without missing information in predictors. All statistical analyses were performed using Stata Version 16.1 (StataCorp LP, College Station, TX, USA).

## Results

### Recruitment and population data

**Fig. 1:**
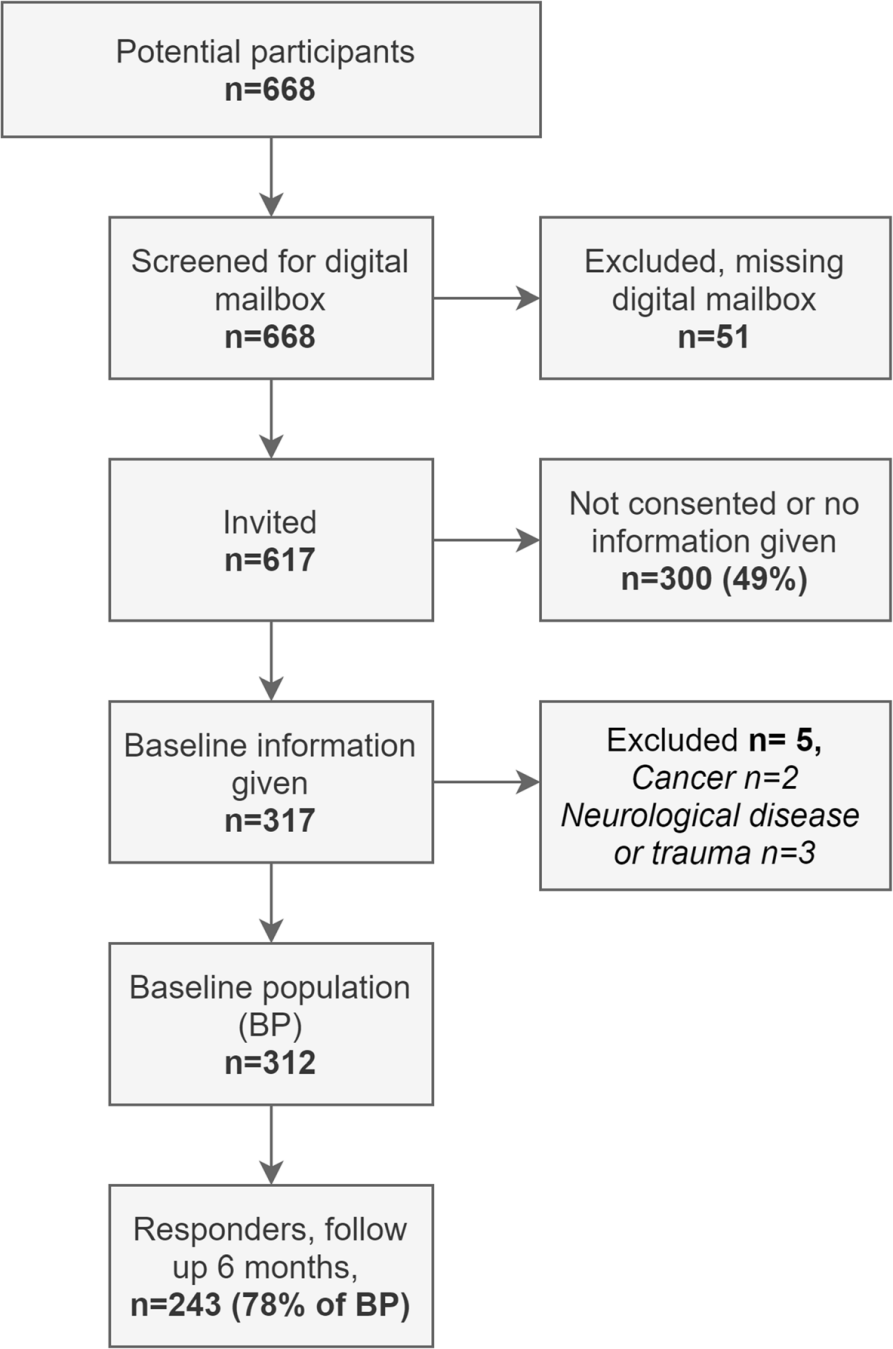
Flow diagram of participant recruitment

In total, 668 patients were referred of which 51 were excluded as they were not registered with a secure digital mailbox. Of the 617 invited patients, 317 agreed to participate. Five of these were excluded due to non-eligible diagnoses; two were referred with cancer and three with neurological diseases. This left 312 patients for the study, of which 243 (78%) returned the six-month follow-up questionnaire (see Fig. 1). Non-responders were younger and less likely to be retired and had a longer duration of symptoms and a poorer self-rated ability to cope with the shoulder pain. Furthermore, they tended to have lower levels of education and poorer mental wellbeing. An additional file describes the differences in more detail (see Additional file 1). Baseline characteristics of included patients (*n* = 312) are shown in Table 2. For responders, the average QuickDASH improvement from first consultation to 6 months follow up was 12.9 (95%CI: 10.4; 15.5).

**Table 2:** Selected summary baseline characteristics of participants (*n* = 312)

Factor	Value
*Sociodemographics*	
Age in years, mean (SD)	54 (14.6)
Female, n (%)	193 (61.9)
Professional educational level, n (%)	
Unskilled	47 (15.0)
Lower level, < 3 years	46 (14.7)
Vocational and training	123 (39.3)
Medium level, > 3–4 years	80 (25.6)
Higher level, > 4 years	16 (5.1)
Employment status, n (%)	
Employed	155 (49.5)
Subsidised employment	18 (5.8)
On leave	2 (.6)
Unemployed	10 (3.2)
Student/under training	15 (4.8)
Retired	99 (31.6)
Other	13 (4.2)
*Clinical characteristics*	
QuickDASH 0–100 score, mean (sd)	41.7 (19.8)
Duration of symptoms in months, mean (sd)	29.4 (52.2)
Median (iqr)	10 (25)
Pain, NPRS 0–10 mean (sd)	5.5 (2.3)
Sick leave, whole or part days^a^, mean (sd)	18.0 (38.0)
Median (iqr)	0 (14)
Movement impairment classification n (%)	
Hypomobility	64 (20.5)
Hypermobility	55 (17.6)
Aberrant motion	193 (61.9)
*Pain behaviour and psychological factors*	
Fear avoidance 0–20 score, mean (sd)	13.7 (7.4)
Pain catastrophizing, 0–20 score, mean (sd)	7.7 (5.4)
Self- rated ability to cope, 0–10 score, mean (sd)	5.9 (2.4)
Self-rated risk of persistent symptoms, 0–10, mean (sd)	5.9 (3.0)
Mental wellbeing, 0–100 score, mean (sd)	56.9 (22.9)
Health-related quality of life, −0,6–1 score, mean (sd)	0.67 (0.15)
Median (iqr)	0.69 (0.16)

Numerical variables with normally distributed data are described by means and standard deviation. Numerical variables with data not normally distributed are described by both means with standard deviations and medians with interquartile ranges

*Abbreviations*: *sd* standard deviation, *iqr* inter quartile range, *NPRS* Numeric pain rating scale, *QuickDASH* Quick Disabilities of the arm, shoulder and hand

^a^Due to current episode of shoulder pain

### Missing values

No candidate predictor, except for sick leave, had more missing values than 3.8% and the distribution appeared random. The following missing values were observed: Professional educational level: 1 (0.3%), baseline QuickDASH: 2 (0.6%), duration of symptoms: 12 (3.8%), sick leave: 103 (33%), fear avoidance: 5 (1.6%), coping: 10 (3.2%), pain catastrophizing: 7 (2.2%), risk of persistent symptoms: 9 (2.9%), mental health: 11 (3.5%), health-related quality of life: 10 (3.2%). In total, 15 patients were not included in the final model due to missing information in predictors.

### Prognostic modelling

The full model explained 30.8% of the variance in QuickDASH change. Parameters are presented in Table 3. After backwards elimination, seven predictors explained 33.3% of the variance: Baseline QuickDASH, employment status, professional educational level, movement impairment classification, self-rated ability to cope with the pain, health-related quality of life and pain catastrophizing. The parameters are presented in Table 4 followed by a practical example on how to calculate predicted change scores. The parsimonious model is presented in Table 5. Further details on model development can be found in Additional file 2. Internal validation by bootstrapping produced a 95% confidence interval of 23 to 43%. The sensitivity analysis using both a best and worst case scenario to include patients lost to follow up (i.e. predicted values +/− MIC), did not change which candidate predictors were included during the model development, with one exception, as age added predictive value in the best case analysis. The supplementary analysis using Akaike’s information criterion instead of the adjusted coefficient of determination led to a slightly different model with duration of symptoms being added to the model. Model performance was marginally better in the model derived from Akaike’s information criterion (*n* = 220). The model performance measures are presented in Table 5 and the parameters from all supplementary analyses are presented in Additional file 3*.*

**Table 3:** Parameters for the full multivariable linear regression model (*n* = 220)

Variable	Coefficient	95% CI	Stand. coefficient
Baseline QuickDASH	0.62	0.41; 0.83	0.60
Age	−0.10	− 0.33; 0.13	− 0.06
Sex			
Woman	Ref.		
Man	−0.39	−5.44; 4.65	− 0.01
Employment status			
Employed/studying	Ref.		
Unemployed/ subsidised employment/ sick leave	−10.89	−18.86; − 2.92	−0.17
Pensioner	1.86	−4.65; 8.38	0.04
Professional educational level			
No education	Ref.		
Low (< 2) or vocational	6.33	−0.57; 13.24	0.15
Medium (3) or high (> 4)	1.81	−5.58; 9.20	0.04
Movement impairment classification			
Hypomobility	Ref.		
Hypermobility	−10.10	−17.60; −2.60	−0.19
Aberrant motion	−8.16	−14.67; − 1.64	− 0.20
Pain	− 0.04	−1.42; 1.34	− 0.005
Duration of symptoms	− 0.05	− 0.10; 0.01	−0.11
Self-rated ability to cope with the pain	1.20	−0.01; 2.41	0.14
Fear avoidance	−0.08	−0.68; 0.52	− 0.02
Health-related quality of life	12.9	−12.6; 38.2	0.09
Mental wellbeing	0.00	−0.14; 0.14	0.00
Self-rated risk of persistent symptoms	0.06	−1.00; 1.11	0.01
Pain catastrophizing	−0.37	−0.95; 0.21	− 0.10
Intercept	−14.28	−42.44; 13.87	–
Adjusted coefficient of determination, adjusted *R*^*2*^ = 30.8% Root MSE^a^ = 17.12			

Positive coefficients reflect a decrease in QuickDASH equal to better function and less symptoms. The model had 17 degrees of freedom, corresponding to 13 participants per degree of freedom

*Abbreviations*: *QuickDASH* Quick Disabilities of the arm, shoulder and hand, *CI* confidence interval

^a^Model standard deviation

**Table 4:** Parameters for the final multivariable linear regression model (*n* = 229)

Variable	Coefficient	95% CI	Stand. coefficient
Baseline QuickDASH	0.61	0.44; 0.78	0.59
Employment status			
Employed/studying	Ref.		
Unemployed/subsidised employment/ sick leave	−11.12	−18.63; − 3.60	−0.17
Pensioner	− 0.22	−5.10; 4.66	− 0.01
Professional educational level			
No education	Ref.		
Low (< 2) or vocational	6.03	−0.42; 12.49	0.15
Medium (3) or high (> 4)	0.94	−5.92; 7.79	0.02
Movement impairment classification			
Hypomobility	Ref.		
Hypermobility	−10.36	−17.44; −3.28	−0.19
Aberrant motion	−8.57	−14.50; −2.63	− 0.21
Self-rated ability to cope with the pain	1.28	0.17; 2.40	0.14
Health-related quality of life^a^	12.1	−10.5; 34.8	0.09
Pain catastrophizing	−0.45	−0.94; 0.04	−0.12
Intercept	−19.91	−42.57; 2.76	–
Adjusted coefficient of determination, adjusted *R*^*2*^ = 33.3% Root MSE^a^ = 16.7			

Positive coefficients reflect a decrease in QuickDASH equal to better function and less symptoms. The model had 10 degrees of freedom, corresponding to 23 participants per degree of freedom

*Abbreviations*: *QuickDASH* Quick Disabilities of the arm, shoulder and hand, *CI* confidence interval

^a^Model standard deviation

**Table 5:** Performance compared over models (*n* = 220)

Model	Adj. R^2^ (95% CI)	AIC value	Root MSE^b^
Final model derived from adj. R^2^	32% (22; 42)	1881	17.0
Final model derived from AIC^a^	32.5% (22;43)	1880	16.9
Parsimonious model using only baseline QuickDASH as predictor	19% (10; 29)	1909	18.47
Model without independent variables	–	–	20.58

*Abbreviations*: *Adj.R*^*2*^ adjusted coefficient of determination, *CI* confidence interval, *AIC* Akaike’s information criterion, *Root MSE* Root mean squared error, *PI* Prediction interval

^a^Duration of symptoms was added in this model along with the predictors from the adj. R2 model

^b^Model standard deviation

The model was designed to calculate the expected improvements for patients at the first consultation so that the treatment trajectory can be adapted from this information.

Model to calculate predicted change in QuickDASH for a given patient:

$$ \Delta  QuickDASH=0.61\times baselineQuickDASH-11.12\times unempl./ subsid./ sick-0.22\times pensioner+6.03\times low/ vocational\ education+0.94\times medium/ high\ education-10.36\times Hypermobility-8.57\times Aberrant\ motion+1.28\times coping+12.1\times HQOL-0.45\times Pain\ catastrophizing-19.91 $$

Note, that categorical variables are scored 0 or 1 depending on which group represents the individual patient. Below are two examples of how to score the model for two different patients with the same baseline QuickDASH (we chose the population mean):

Patient A characteristics: Baseline QuickDASH = 42. Employment status = Studying. Professional educational level = Low. Movement impairment classification = Hypomobility. Self-rated ability to cope with the pain = 9. Health-related quality of life = 0.9. Pain catastrophizing = 3.

Patient B characteristics: Baseline QuickDASH = 42. Employment status = Unemployed. Professional educational level = None. Movement impairment classification = Aberrant motion. Self-rated ability to cope with the pain = 2. Health-related quality of life = 0.4. Pain catastrophizing = 17.

$$ \Delta  QuickDASH\left( patient\ A\right)=0.61\times \mathbf{42}-11.12\times \mathbf{0}-0.22\times \mathbf{0}+6.03\times \mathbf{1}+0.94\times \mathbf{0}-10.36\times \mathbf{0}-8.57\times \mathbf{0}+1.28\times \mathbf{9}+12.1\times \mathbf{0}.\mathbf{9}-0.45\times \mathbf{3}-19.91=32.8 $$

$$ Calculation\ of\ 95\% prediction\ interval=32.8\pm 1.96\times RootMSE=32.8\pm 1.96\times 16.7=0.1;65.5 $$

$$ \Delta  QuickDASH\left( patient\ B\right)=0.61\times \mathbf{42}-11.12\times \mathbf{1}-0.22\times \mathbf{0}+6.03\times \mathbf{0}+0.94\times \mathbf{0}-10.36\times \mathbf{0}-8.57\times \mathbf{1}+1.28\times \mathbf{2}+12.1\ast \mathbf{0}.\mathbf{4}-0.45\times \mathbf{1}\mathbf{7}-19.91=-14.2 $$

$$ Calculation\ of\ 95\% prediction\ interval=-14.2\pm 1.96\times RootMSE=-14.2\pm 1.96\times 16.7=-46.9;18.5 $$

Thus, from first consultation to 6 months follow up, patients with the characteristics of patient A are expected to gain a 33-point QuickDASH improvement (95%PI: 0; 66) while patients with the characteristics of patient B are expected to experience worse symptoms and function corresponding to a rise of 14 points on the QuickDASH (95% PI: − 47; 19) despite treatment. In this case, patient A could continue the planned course of physiotherapy while patient B probably would need other or supplementary treatment in order to improve. In more moderate cases, e.g. an expected change of 12 points, the clinician would have to judge whether this improvement would be sufficient to continue usual treatment in coherence with the patient. This judgement could depend on the choice of minimal clinical important difference threshold which ranges from 8 to 16 depending on methodology, setting and subpopulation [60, 61] and the patient’s motivation for treatment etc. Thus, the numerical nature of this prognostic model allows for more individual judgement in each patient-case. However, the uncertainty of the predictions reflected in the root MSE and the 95% prediction intervals must be noted.

## Discussion

### Principal findings and comparison with existing literature

The final prognostic model explained 33.3% of the variance in QuickDASH change scores with a root mean squared error of 16.7. It included seven predictors; baseline QuickDASH score, employment status, professional educational level, movement impairment classification, self-rated ability to cope with the pain, health-related quality of life and pain catastrophizing.

In line with previous research, we found higher baseline disability and symptoms to be associated with greater improvement, whereas higher pain catastrophizing, lower educational level and unemployment/sick leave/working a subsidised job was associated with less improvement [8, 62]. As previous literature, we found limited predictive value of sex [62].

In contrast to other studies, we found self-rated ability to cope with the pain to be associated with larger improvement, while age, pain (NPRS), mental wellbeing and fear avoidance showed limited predictive value [8, 15]. These differences might be explained by variation in design, outcome measures, inclusion criteria, setting and by the fact that most previous studies included patients with shorter duration of symptoms than of those in the present study.

Our model included two predictors that have not been examined in shoulder patients before. Although not reaching statistical significance, health-related quality of life explained a noteworthy amount of the variance in the QuickDASH change scores. Furthermore, movement impairment classification had a strong association with QuickDASH change scores in our model, where hypomobility was associated with a greater improvement than hypermobility and aberrant motion. To our knowledge, such diagnostic classifications have not previously been shown predictive in shoulder patients [19]. Thus, movement impairment classification should be examined in future prognostic studies. The relevance of our prediction model was supported by the fact that it consisted of biological, psychological and social factors and it was therefore theoretically in line with the main categories of the biopsychosocial model [63].

### Strengths and limitations

The prospective design adds to the strengths of this study along with the rigorous reporting of the prognostic modelling which allows replication of the study methods (Additional file 2). Also, we based the elimination process on presumptions from clinical rationale and the literature, rather than on conventional significance levels [13].

In a sensitivity analysis, we examined the possible impact of potential attrition bias since 22% the included participants did not provide outcome data at 6 months. The sensitivity analysis showed that the model did not change when extreme values were assigned to the dropouts except in a best-case scenario where age was included. Thus, the model was robust to such potential attrition biases, but age could possibly hold prognostic value depending on the true change scores of the dropouts. A supplementary analysis using Akaike’s information criterion for backwards elimination instead of the adjusted coefficient of determination led to a model including duration of symptoms. Thus, duration of symptoms could potentially hold prognostic value in the model (resulting in marginal improvement in model performance). As we had 13 participants per degree of freedom in the full model, our sample size was in line with suggested rules of thumb of having more than ten participants per degree of freedom [11, 56]. However, a larger sample size would have been preferable and could potentially lead to differences in the model.

The use of self-reported data could potentially have caused information problems since patients might knowingly or unknowingly have reported themselves as better or worse than they were [64, 65]. Since this study was prospective, patients did not have knowledge of their outcome when reporting potential predictors. Thereby, it is unlikely that potential information problems in the predictors are dependent on the outcome variable, which minimizes the risk of bias. However, there is a risk of information bias, since it was not possible to blind participants to prognostic factors at the outcome assessment whereby reporting of outcome could be dependent on the predictors [11]. However, the risk is probably low since the patients did not have knowledge about the studied associations and there were 6 months between predictor- and outcome assessment. Further, we used a brief version of the pain catastrophizing scale along with subscales of the Örebro Musculoskeletal pain questionnaire, which might have affected the ability to capture the constructs of interest and their importance as predicters. However, the brief version of the pain catastrophizing scale has previously been validated [45] and the Örebro Musculoskeletal subscales were developed to capture the constructs of interest and have been validated in brief forms [40, 41].

We did not attempt to correct or impute, as missing values in candidate predictors were few and the distribution appeared random. Although we included a range of candidate predictors, other variables could have been included such as biomarkers, occupational factors, lifestyle factors and a series of other psychological factors [8, 13]. Furthermore, information might have been lost when grouping the variables “professional educational level” and “employment status” into bigger groups. Also, a model accounting for interaction between variables could potentially have led to a different final prediction model. However, it was chosen not to consider interactions between variables since this study is not examining causal pathways [11, 66]. Further, additional diagnostics and clinical tests could have been considered as candidate predictors, but as their performance, interpretability and information on diagnosis and prognosis is limited, such data was not collected in the present study [67–69].

We used a 6-month follow up though it could be argued that a follow-up period of 12 months would be preferable. However, 6 months was chosen, since treatment rarely exceeds this period of time, and previous studies of common musculoskeletal disorders shows that only little additional change happens beyond 6 months [70].

When using our results, it should be noted that this was an explorative multivariable study aiming to develop a prognostic model and not to explore individual factors, and therefore the parameters do not represent the factors’ independent contribution to the prediction [14, 71]. Also, our population had a mean duration of symptoms of 29 months, and therefore our findings are probably not applicable to shoulder conditions of shorter duration as seen in primary care settings. Further, the modest recruitment rate could affect the generalisability of our findings, since only half of the invited patients agreed to participate.

When comparing models (for *n* = 220), the root mean squared error only dropped from 20.6 in the model without independent variables to 17 in the final model, meaning that the residuals were only moderately less spread out in the final model. The final model explained 13% more of the variance in QuickDASH change than a model predicting change from only baseline QuickDASH. We believe that the collection of these extra variables contributing with 13% extra explained variance is justified by the fact that they are easy to collect along with QuickDASH scores. However, the model performance was modest, and the root mean squared error (model SD) was large meaning that the predictions were quite imprecise. Thus, there is limited clinical utility of the model in its current form on an individual prediction level. Therefore, possible contributions of other predictors should be explored in order to optimise the performance and preciseness of the model while a larger sample sizes could be considered.

By presenting a combination of variables with predictive ability, we hope that our study can guide future work on developing a model that can be used as a tool for stratified care. With such a tool, e.g. patients predicted to have satisfactory change scores at baseline can receive usual care, while patients predicted to have lower change scores can be directed to more intensive and specialised care options.

## Conclusion

In this study, we aimed to develop a prognostic model to inform clinical decision making in order to improve outcomes for patients with persistent shoulder pain referred to physiotherapy rehabilitation. However, the precision of the predictions on an individual patient level was low. The final prognostic model included seven predictors explaining 33% of the variance in QuickDASH change with a root mean squared error (model SD) of 17 points. Two of the included predictors were novel: Movement impairment classification based on diagnosis and health-related quality of life. Further work needs to be done in order to optimise the prognostic model including exploration of the contribution of other potential predictors before external validity can be examined along with the potential impact of the model in clinical practice. Thus, in its current form our model is more suitable for guiding future predictive work than for guiding clinical decision making.

## Supplementary Information

**Additional file 1.** NON-RESPONDER ANALYSIS.

**Additional file 2.** MODEL DEVELOPMENT REPORT.

**Additional file 3.** SUPPLEMENTARY ANALYSES.

## Data Availability

The dataset used and analysed during the current study is available from the corresponding author on reasonable request.

## Abbreviations

QuickDASH: Quick Disabilities of the Arm, Shoulder and Hand
WHO-5: WHO mental wellbeing index
NPRS: Numeric pain rating scale
MIC: Minimal important change
Adjusted R^2^: Adjusted coefficient of determination
