# Correction to: Predicting change in symptoms and function in patients with persistent shoulder pain: a prognostic model development study

**DOI:** 10.1186/s12891-021-04724-5

**Published:** 2021-10-08

**Authors:** Mathias Moselund Rønnow, Thor André Brøndberg Stæhr, David Høyrup Christiansen

**Affiliations:** Department of Occupational Medicine, University Research Clinic, Regional Hospital West Jutland, Gl. Landevej 61, 7400 Herning, Denmark

**Correction to: BMC Musculoskelet Disord 22, 732 (2021)**

**https://doi.org/10.1186/s12891-021-04612-y**

Following the publication of the original article [1] the authors noticed that the statement “all authors contributed equally” appeared in the published article. This statement is directly opposite of the author contribution statement in the article. The original article [1] has been updated.
