# Supplementary material for: Predicting change in symptoms and function in patients with persistent shoulder pain: a prognostic model development study
Source: BMC Musculoskelet Disord. 2021 Aug 27;22:732. doi: 10.1186/s12891-021-04612-y (PMC8401246; doi:10.1186/s12891-021-04612-y)
Supplement: Supplementary file 1 — Additional file 1. NON-RESPONDER ANALYSIS. [file 12891_2021_4612_MOESM1_ESM.pdf]

ADDITIONAL FILE 1 –NON-RESPONDER ANALYSIS

Rønnow MM, Stæhr ABS and Christiansen DH "Predicting change in symptoms and function in patients with persistent shoulder pain: A prognostic model development study". *BMC Musculoskeletal Disorders* 2021.

Table S1. Differences between responders and non-responders at 6 months follow-up.

**Factor**	**Responders n=243**	**Non-responders n=69**	**Difference**
Numerical variables			Mean [CI] or P-value*
Age (years), mean (sd)	56.3 (13.6)	45.4 (14.8)	**-10.9 [-14.6; -7.2]**
QuickDASH, mean (sd)	41.7 (20.2)	41.8 (18.3)	0.1 [-5.1; 5.5]
Duration of symptoms (months), median (iqr)<br>mean (sd)	*7 (22)*<br>26.6 (51.3)	*17 (47)*<br>39.1 (54.8)	***P<0.005***
Pain (typical last 14 days), mean (sd)	5.4 (2.3)	5.7 (2.1)	0.3 [-0.3; 0.9]
Sick leave** (whole or part days), median (iqr)<br>mean (sd)	*1 (16)*<br>18.8 (37.6)	*0 (7)*<br>15.9 (39.4)	*P=0.31*
Fear avoidance, mean (sd)	13.8 (7.9)	13.4 (4.9)	-0.4 [-2.3; 1.6]
Pain catastrophizing, mean (sd)	7.5 (5.4)	8.4 (5.6)	0.9 [-0.5; 2.4]
Self-rated ability to cope with pain, mean (sd)	6.1 (2.3)	5.4 (2.7)	**-0.7 [-1.3; -0.1]**
Self-rated risk of persistent symptoms, mean (sd)	5.8 (3.0)	6.1 (2.9)	0.3 [-0.5; 1.1]
Mental wellbeing, mean (sd)	58.0 (23.7)	53.4 (19.9)	-4.4 [-10.8; 1.6]
Health-related quality of life, median (iqr)<br>mean (sd)	*0.71 (0.15)*<br>*0.67 (15)*	*0.68 (0.14)*<br>*0.66 (0.13)*	*P=0.29*
Categorical variables			%-points [CI]
Sex (female), n (%)	148 (61.0)	45 (65.2)	4.2 [-8.5; 17.1]
Professional educational level, n (%)			
Unskilled	36 (14.8)	11 (15.9)	1.1 [-10.9; 8.5]
Lower level (< 3 years)	33 (13.6)	13 (18.8)	5.2 [-4.9; 15.5]
Vocational and training	91 (37.5)	31 (44.9)	7.4 [-5.7; 20.7]
Medium level (>3-4 years)	68 (28.0)	12 (17.4)	-10.6 [-21.0; 0.02]
Higher level (>4 years)	14 (5.8)	2 (2.9)	-2.9 [-7.8; 2.1]
Employment status, n (%)			
Employed	118 (48.6)	37 (53.6)	5.0 [-8.3; 18.4]
Employed, special terms	11 (4.5)	7 (10.1)	5.6 [-1.9; 13.2]
On leave	2 (0.8)	0	-0.8 [-1.9; 0.3]
Unemployed	7 (2.9)	3 (4.4)	1.5 [-3.8; 6.7]
Student/under training	10 (4.1)	5 (7.2)	3.1 [-3.5; 9.7]
Retired	86 (35.4)	13 (18.8)	**-16.6 [-27.6; -5.5]**
Other	9 (3.7)	4 (5.8)	2.1 [-3.9; 8.1]
Movement impairment classification, n (%)			
Hypomobility	53 (21.8)	11 (15.9)	-5.9 [-15.9; 4.2]
Hypermobility	42 (17.3)	13 (18.8)	1.5 [-8.8; 11.9]
Aberrant motion	148 (60.9)	45 (65.2)	4.3 [-8.5; 17.1]

* Tested by Wilcoxon rank-sum test (not normally distributed variables). ** Due to current episode of shoulder pain. Numbers may vary due to missing values, and percentages may not add completely to 100% due to rounding of numbers. Abbreviations: CI= confidence intervals, sd=standard deviation, iqr=interquartile range QuickDASH= Quick Disabilities of the arm, shoulder and hand.
