# Supplementary material for: Predicting change in symptoms and function in patients with persistent shoulder pain: a prognostic model development study
Source: BMC Musculoskelet Disord. 2021 Aug 27;22:732. doi: 10.1186/s12891-021-04612-y (PMC8401246; doi:10.1186/s12891-021-04612-y)
Supplement: Supplementary file 2 — Additional file 2. MODEL DEVELOPMENT REPORT. [file 12891_2021_4612_MOESM2_ESM.pdf]

ADDITIONAL FILE 2 – MODEL DEVELOPMENT REPORT

Rønnow MM, Stæhr ABS and Christiansen DH "Predicting change in symptoms and function in patients with persistent shoulder pain: A prognostic model development study". *BMC Musculoskeletal Disorders* 2021.

The aim of this appendix is to report all steps in the development process of the prognostic model to clarify how variables were eliminated and on what basis. We wanted a model suitable for clinical practice and believe that it should not be too comprehensive since this will result in too high a time consumption and burden for both the patient and the clinician. Therefore, a "full model" including all relevant measured variables was pruned using a modified stepwise backwards elimination. The development process is shown below. The full model, from which backwards eliminations was performed, was deemed in line with the assumptions of multiple linear regression and had the following parameter estimates:

Table S2 Parameters for the full multivariable linear regression model (n=220).

Variable	Coefficient	95% CI	p-value	Stand. coef
Baseline QuickDASH	0.62	0.41; 0.83	<0.001	0.60
Age	-0.10	-0.33; 0.13	0.40	-0.06
Sex				
Woman	Ref.			
Man	-0.39	-5.44; 4.65	0.89	-0.01
Employment status				
Employed/studying	Ref.			
Unemployed/Special terms/ sick leave	-10.89	-18.86; -2.92	<0.01	-0.17
Pensioner	1.86	-4.65; 8.38	0.57	0.04
Professional educational level				
No education	Ref.			
Low (<2) or vocational	6.33	-0.57; 13.24	0.07	0.15
Medium (3) or high (>4)	1.81	-5.58; 9.20	0.63	0.04
Movement impairment classification				
Hypomobility	Ref.			
Hypermobility	-10.10	-17.60; -2.60	<0.01	-0.19
Aberrant motion	-8.16	-14.67; -1.64	0.014	-0.20
Pain	-0.04	-1.42; 1.34	0.95	-0.005
Duration of symptoms	-0.05	-0.10; 0.01	0.09	-0.11
Self-rated ability to cope with the pain	1.20	-0.01; 2.41	0.05	0.14
Fear avoidance	-0.08	-0.68; 0.52	0.80	-0.02
Health-related quality of life	12.9	-12.6; 38.2	0.32	0.09
Mental wellbeing	0.00	-0.14; 0.14	1	0.00
Self-rated risk of persistent symptoms	0.06	-1.00; 1.11	0.91	0.01
Pain catastrophizing	-0.37	-0.95; 0.21	0.2	-0.10
Intercept	-14.28	-42.44; 13.87	0.32	-
Adjusted coefficient of determination, adjusted R^2 = 30.8%				

Positive coefficients reflect a decrease in QuickDASH equal to better function and less symptoms. The model had 17 degrees of freedom, corresponding to 13 participants per degree of freedom. Abbreviations: QuickDASH= Quick Disabilities of the arm, shoulder and hand, CI =confidence interval.

Variables were eliminated by:

1. Holding up sets of variables against each other by presumptions on overlap in predictor information (based on the literature and clinical reasoning(1)). It was checked whether the presumed interrelated variables were correlated using Spearman´s correlation, since high correlations indicate that one of the variables does not add much to the prediction(2). For a variable to be eliminated, it could not lead to a drop in the adjusted coefficient of determination (adjusted R^2). This step was chosen before conventional backwards elimination since the candidate predictors were not specifically collected for prognostic modelling.
2. When no more presumptions were present, standardized coefficients, correlations and p-values were used to indicate the next variables to be eliminated(3). Deletion of a variable could not lead to a drop in the adjusted R^2 (drops under 0.5% were however allowed).

The following was presumed:

1. In the EQ5D, the patients are asked questions regarding pain, function and anxiety/depression. Therefore it was presumed that health-related quality of life (EQ5D) could explain changes in QuickDASH for both pain (NRPS) and mental wellbeing (WHO-5) (more pain and lower mental wellbeing leading to lower health-related quality of life)(4).
2. Pain catastrophizing is characterized by the tendency to magnify the threat value of pain and to feel helpless in the context of pain and by a relative inability to inhibit pain-related thoughts in anticipation of, during or following pain(5). Therefore, it was presumed that people with higher levels of pain catastrophizing would see their shoulder pain as more likely to become persistent.
3. It was presumed that self-rated ability to cope with the shoulder pain would be contained in or strongly connected to pain catastrophizing (from the characterization of pain catastrophizing: "..to feel helpless in the context of pain..")(5, 6).
4. It was presumed that fear avoidance was connected to pain catastrophizing and self-rated ability to cope with the pain since pain catastrophizing and a feeling of not being able to cope with the pain, could lead to fear avoidance(6).

Steps in the elimination:

Elimination was performed from the previous step (e.g. elimination in step two was performed on the model advancing from step one).

1. Presumption 1: There was a high correlation between health-related quality of life and mental wellbeing (0.62) and a high negative correlation between health-related quality of life and pain (-0.55), supporting the presumption. Firstly, mental wellbeing was eliminated from the model, leading to a higher adjusted R^2. Then pain was eliminated, leading to a further rise in the adjusted R^2. A model with both pain and mental wellbeing instead of health-related quality of life was examined to assess whether this would lead to a higher adjusted R^2 than the model with health-related quality of life. However, this led to an adjusted R^2 close to that of the baseline model= 30.8%.
   Eliminated in step 1: mental wellbeing and pain (adjusted R^2 = 31.8%)
2. Presumption 2: There was a correlation between the two variables of 0.55, supporting the presumption. Self-rated risk of persistent symptoms was eliminated, resulting in a rise in adjusted R^2. Eliminating pain catastrophizing instead, would lead to a high drop in adjusted R^2.
   Eliminated in step 2: Self-rated risk of persistent symptoms (adjusted R^2 = 32.2%)
3. Presumption 3: Self-rated ability to cope with the pain was moderately negatively correlated with pain catastrophizing (-0.43). Self-rated ability to cope with the pain was eliminated, but this led to a drop in adjusted R^2. Therefore, self-rated ability to cope with the pain was kept in the model. Eliminating pain catastrophizing instead would lead to a higher drop in adjusted R^2.
   Eliminated in step 3: None
4. Presumption 4: Fear avoidance was eliminated, leading to a rise in adjusted R^2. Eliminating pain catastrophizing or self-rated ability to cope with the pain instead of fear avoidance led to a drop in adjusted R^2. Fear avoidance was only correlated with pain catastrophizing by 0.42 and with self-rated ability to cope with the pain by -0.18, which did not support the presumption. However, fear avoidance was correlated with baseline QuickDASH by 0.57 and health-related quality of life by -0.48, which might explain why it could be deleted without loss in the adjusted R^2.

Eliminated in step 4: Fear avoidance (adjusted R^2 = 32.5%)

5. After no more presumptions were present, p-values, standardized betas and correlations were assessed in order to determine the next variable to be eliminated. As mentioned, adjusted R^2 could not drop by more than 0.5% and the following variables were eliminated:
   a. Age.
   b. Sex.
   c. Duration of symptoms.

   This led to a rise in adjusted R^2 = 33.3%.

The final model:

ΔQuickDASH dependant on: Baseline QuickDASH + employment status (3) + professional educational level (3) + MSDC (3) + self-rated ability to cope with the pain + health-related quality of life + pain catastrophizing.

The final model was also deemed to fulfil the assumptions of multiple linear regression. The parameter estimates are shown in Table S3.

Table S3 Parameters for the final multivariable linear regression model (n=229)

**Variable**	**Coefficient**	**95% CI**	**p-value**	**Stand. Coef**
Baseline QuickDASH	0.61	0.44; 0.78	<0.001	0.59
Employment status				
Employed/studying	Ref.			
Unemployed/Special terms/ sick leave	-11.12	-18.63; -3.60	<0.005	-0.17
Pensioner	-0.22	-5.10; 4.66	0.93	-0.01
Professional educational level				
No education	Ref.			
Low (<2) or vocational	6.03	-0.42; 12.49	0.07	0.15
Medium (3) or high (>4)	0.94	-5.92; 7.79	0.79	0.02
Movement impairment classification				
Hypomobility	Ref.			
Hypermobility	-10.36	-17.44; -3.28	<0.005	-0.19
Aberrant motion	-8.57	-14.50; -2.63	<0.005	-0.21
Self-rated ability to cope with the pain	1.28	0.17; 2.40	<0.05	0.14
Health-related quality of life	12.1	-10.5; 34.8	0.29	0.09
Pain catastrophizing	-0.45	-0.94; 0.04	0.072	-0.12
Intercept	-19.91	-42.57; 2.76	0.09	-
Adjusted coefficient of determination, adjusted R^2 = 33.3%				

Positive coefficients reflect a decrease in QuickDASH equal to better function and less symptoms. The model had 10 degrees of freedom, corresponding to 23 participants per degree of freedom. Abbreviations: QuickDASH= Quick Disabilities of the arm, shoulder and hand, CI =confidence interval.

Plots:

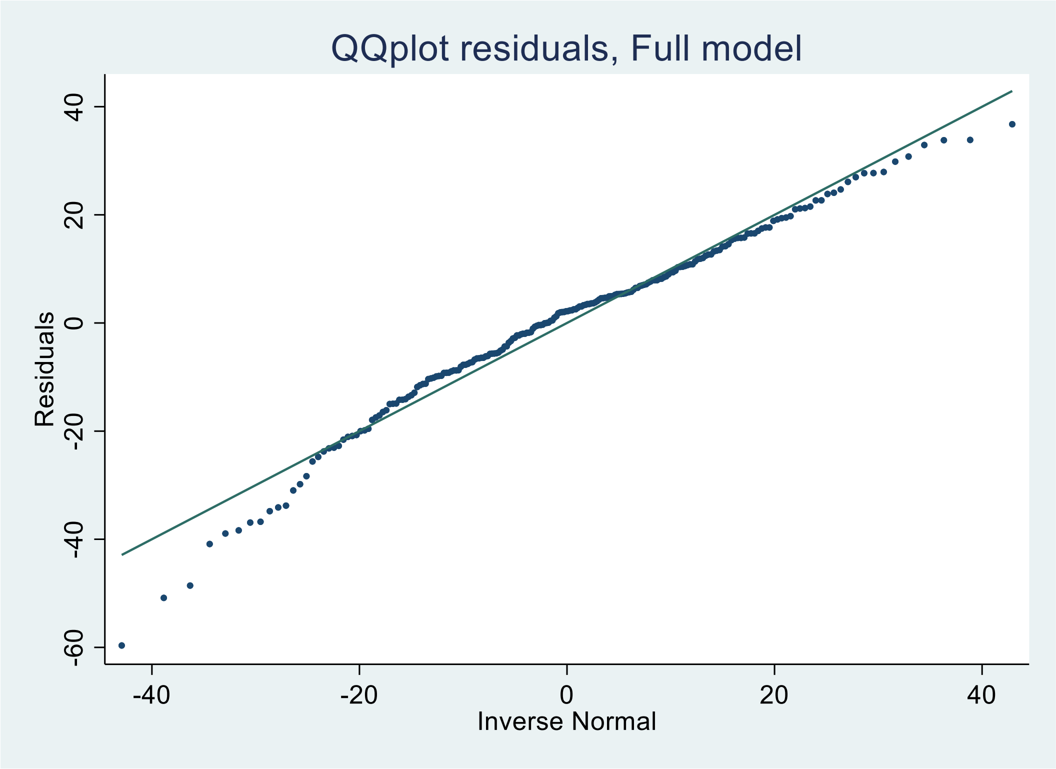

QQplot residuals, Full model
Residuals
-60
-40
-20
0
20
40
-40
-20
0
20
40
Inverse Normal

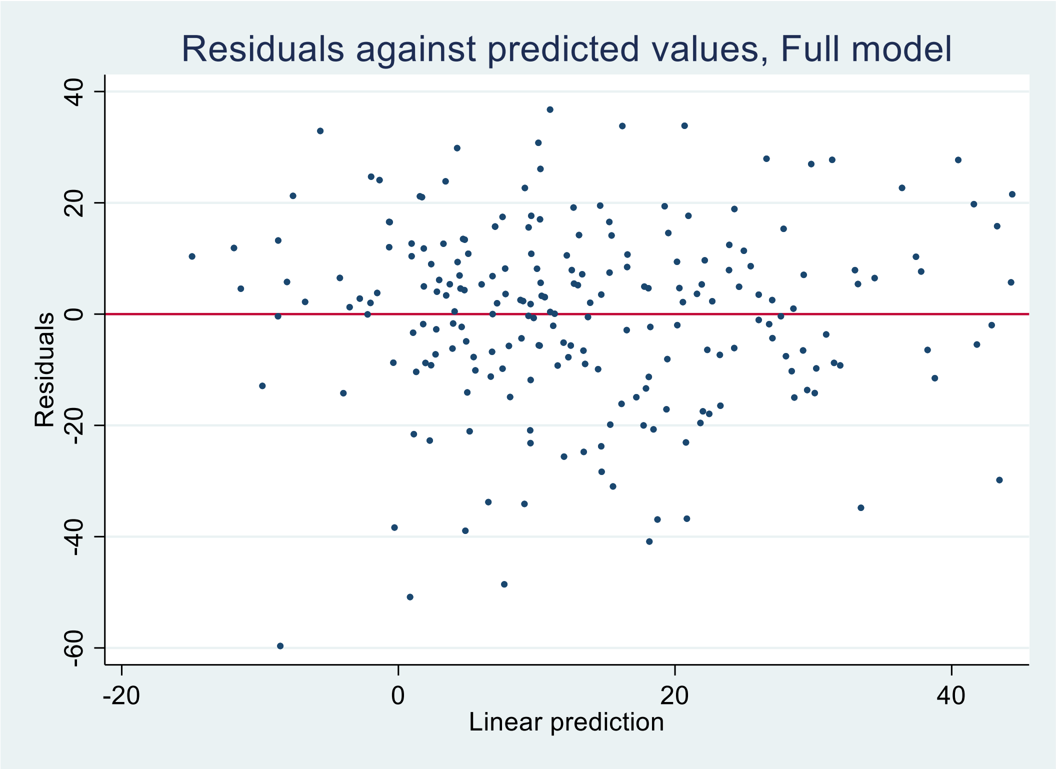

Residuals against predicted values, Full model
Residuals
-60
-40
-20
0
20
40
-20
0
20
40
Linear prediction

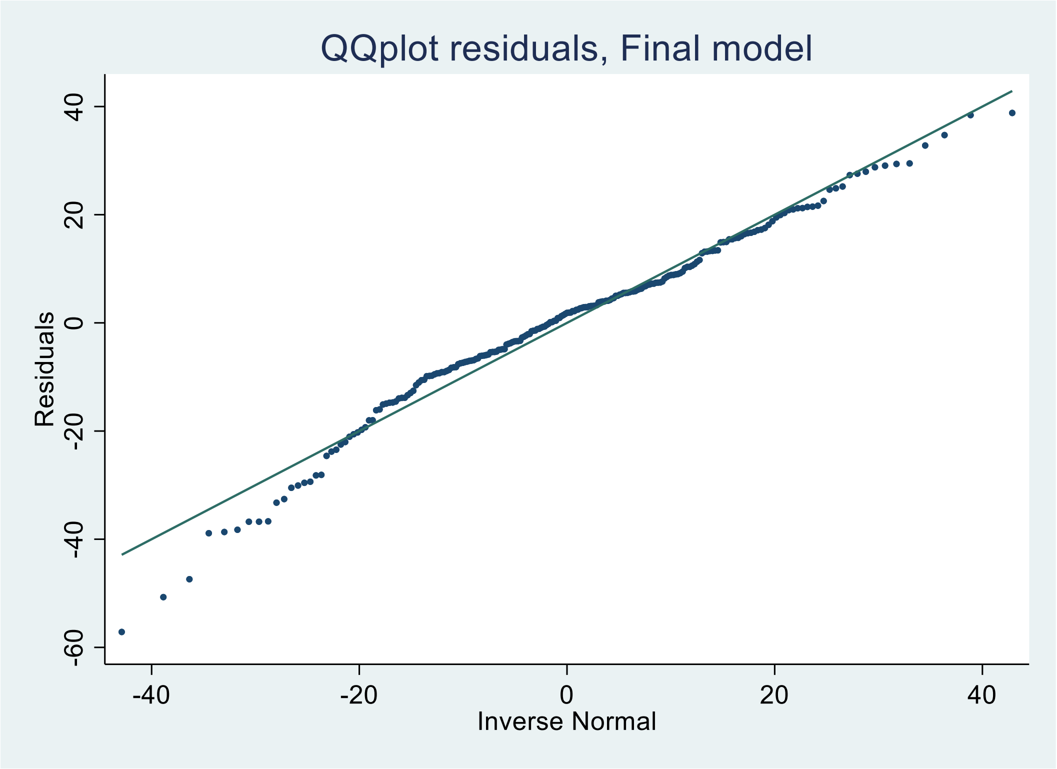

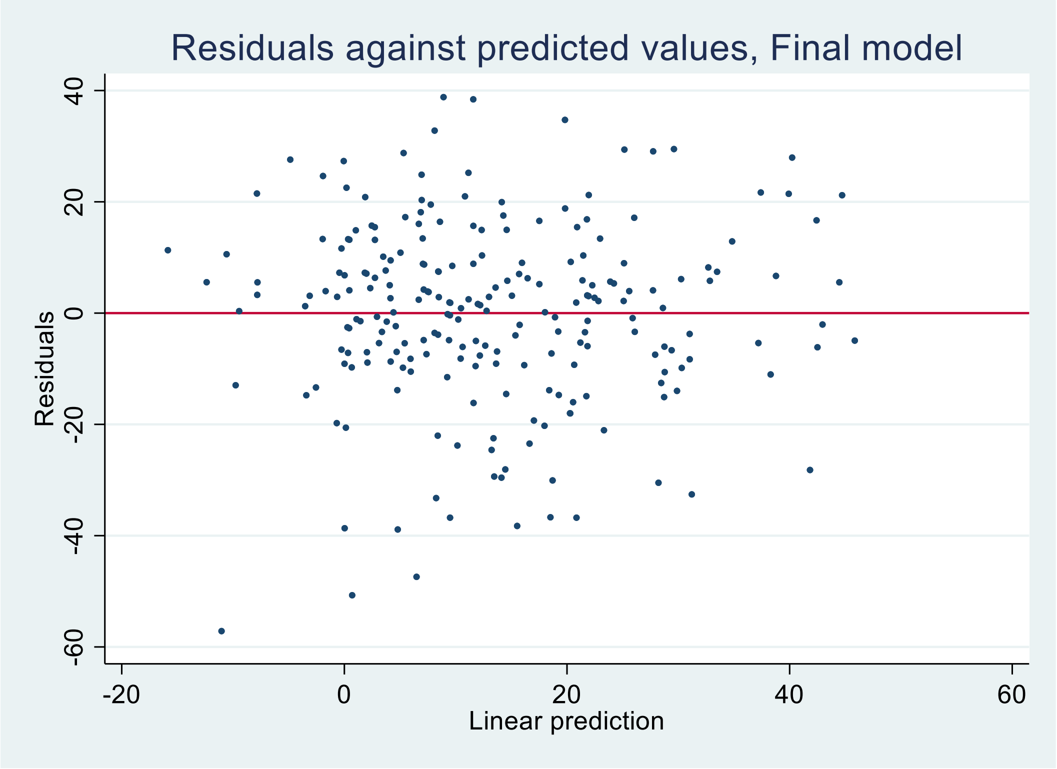

1. Moons KGM, Altman DG, Reitsma JB, Ioannidis JPA, Macaskill P, Steyerberg EW, et al. Transparent Reporting of a multivariable prediction model for Individual Prognosis

Or Diagnosis (TRIPOD): Explanation and Elaboration. Annals of Internal Medicine. 2015;162(1):W1-W73.
2. Royston P, Moons KG, Altman DG, Vergouwe Y. Prognosis and prognostic research: Developing a prognostic model. Bmj. 2009;338:b604.
3. Royston P. Multivariable model-building: a pragmatic approach to regression analysis based on fractional polynomials for modelling continuous variables. Sauerbrei W, editor. Hoboken, N.J. : Chichester: Wiley ; John Wiley [distributor]; 2008.
4. Organization WH. Investing in mental health. Geneva: World Health Organization; 2003.
5. Quartana PJ, Campbell CM, Edwards RR. Pain catastrophizing: a critical review. Expert review of neurotherapeutics. 2009;9(5):745-58.
6. Linton SJ, Shaw WS. Impact of Psychological Factors in the Experience of Pain. Physical therapy. 2011;91(5):700-11.
