# Supplementary material for: Predicting change in symptoms and function in patients with persistent shoulder pain: a prognostic model development study
Source: BMC Musculoskelet Disord. 2021 Aug 27;22:732. doi: 10.1186/s12891-021-04612-y (PMC8401246; doi:10.1186/s12891-021-04612-y)
Supplement: Supplementary file 3 — Additional file 3. SUPPLEMENTARY ANALYSES. [file 12891_2021_4612_MOESM3_ESM.pdf]

ADDITIONAL FILE 3 – SUPPLEMENTARY ANALYSES

Rønnow MM, Stæhr ABS and Christiansen DH "Predicting change in symptoms and function in patients with persistent shoulder pain: A prognostic model development study". *BMC Musculoskeletal Disorders 2021*.

BEST-CASE AND WORST-CASE SENSITIVITY ANALYSES

The model development is identical to the one described in appendix 2, "Model development report". Therefore, only the parameter estimates of the full and the final model are presented for this analysis.

Table S4 Parameters for the full best-case model (n=283)

**Variable**	**Coefficient**	**95% CI**	**p-value**	**Stand. Coef.**
Baseline QuickDASH	0.60	0.43; 0.76	<0.001	0.60
Age	-0.25	-0.42; -0.08	0.005	-0.18
Sex				
Woman	Ref.			
Man	-0.73	-4.85; 3.39	0.73	-0.02
Employment status				
Employed/studying	Ref.			
Unemployed/Special terms/ sick leave	-9.92	-15.96; -3.87	0.001	-0.17
Pensioner	3.50	-1.95; 8.96	0.21	0.08
Professional educational level				
No education	Ref.			
Low (<2) or vocational	7.38	1.72; 13.03	0.01	0.19
Medium (3) or high (>4)	1.22	-4.97; 7.41	0.70	0.03
Movement impairment classification				
Hypomobility	Ref.	Ref.		
Hypermobility	-10.32	-16.92; -3.93	<0.005	-0.20
Aberrant motion	-8.85	-14.38; -3.31	<0.005	-0.22
Pain	-0.01	-1.18; 1.15	0.98	-0.00
Duration of symptoms	-0.03	-0.07; 0.01	0.11	-0.09
Self-rated ability to cope with the pain	1.05	0.12; 1.97	<0.05	0.13
Fear avoidance	-0.06	-0.54; 0.42	0.81	-0.02
Health related quality of life	13	-7; 34	0.21	0.10
Mental wellbeing	-0.01	-0.13; 0.12	0.93	-0.01
Self-rated risk of persistent symptoms	0.02	-0.83; 0.87	0.96	0.00
Pain catastrophizing	-0.40	-0.87; 0.08	0.10	-0.11
Intercept	-1.87	-24.04; 20.31	0.87	-
Adjusted coefficient of determination, adjusted R^2 = 34.4%				

Positive coefficients reflect a decrease in QuickDASH equal to better function and less symptoms. The model had 17 degrees of freedom, corresponding to 16 patients per degree of freedom. Abbreviations: QuickDASH= Quick Disabilities of the arm, shoulder and hand, CI =confidence interval.

Table S5 Parameter estimates for final best-case model (n=292)				
**Variable**	**Coefficient**	**95% CI**	**p-value**	**Stand. Coef.**
Baseline QuickDASH	0.60	0.46; 0.73	<0.001	0.60
Age	-0.24	-0.41; -0.08	<0.005	-0.18
Employment status				
Employed/studying	Ref.			
Unemployed/subsidised employment/ sick leave	-10.75	-16.61; -4.89	<0.001	-0.18
Pensioner	2.86	-2.29; 8.02	0.28	0.07
Professional educational level				
No education	Ref.			
Low (<2) or vocational	7.24	1.87; 12.61	0.01	0.18
Medium (3) or high (>4)	0.48	-5.36; 6.33	0.87	0.01
Movement impairment classification				
Hypomobility	Ref.			
Hypermobility	-11.20	-17.29; -5.11	<0.001	-0.22
Aberrant motion	-9.60	-14.75; -4.45	<0.001	-0.24
Self-rated ability to cope with the pain	1.13	0.26; 2.0	<0.05	0.14
Health-related quality of life	12	-6; 31	0.18	0.09
Pain catastrophizing	-0.43	-0.83; -0.03	<0.05	-0.12
Intercept	-3.51	-23.35; 16.32	0.73	
Adjusted coefficient of determination, adjusted R^2 = 36%				

Positive coefficients reflect a decrease in QuickDASH equal to better function and less symptoms. The model had 11 degrees of freedom, corresponding to 26 patients per degree of freedom. Abbreviations: QuickDASH= Quick Disabilities of the arm, shoulder and hand, CI =confidence interval.

Table S6, parameter estimates for the full worst-case model

Variable	Coefficient	95% CI	p-value	Stand. Coef.
Baseline QuickDASH	0.64	0.47; 0.80	<0.001	0.62
Age	0.07	-0.12; 0.23	0.5	0.04
Sex				
Woman	Ref.			
Man	-0.06	-4.18; 4.06	0.98	-0.00
Employment status				
Employed/studying	Ref.			
Unemployed/Special terms/ sick leave	-11.85	-17.90; -5.81	<0.001	-0.19
Pensioner	0.23	-5.23; 5.68	0.94	0.01
Professional educational level				
No education	Ref.			
Low (<2) or vocational	5.29	-0.36; 10.95	0.07	0.13
Medium (3) or high (>4)	2.40	-3.79; 8.59	0.45	0.05
Movement impairment classification				
Hypomobility	Ref.	Ref.		
Hypermobility	-9.87	-16.27; -3.48	<0.005	-0.19
Aberrant motion	-7.46	-13.01; -1.93	<0.01	-0.18
Pain	-0.07	-1.24; 1.10	0.91	-0.01
Duration of symptoms	-0.06	-0.10; -0.02	<0.01	-0.15
Self-rated ability to cope with the pain	1.36	0.43; 2.28	<0.01	0.16
Fear avoidance	-0.09	-0.57; 0.38	0.70	-0.02
Health related quality of life	12	-8; 33	0.24	0.09
Mental wellbeing	0.01	-0.12; 0.13	0.93	0.01
Self-rated risk of persistent symptoms	0.10	-0.75; 0.94	0.83	0.01
Pain catastrophizing	-0.34	-0.81; 0.13	0.16	-0.09
Intercept	-26.70	-48.87; -4.52	<0.05	-
Adjusted coefficient of determination, adjusted R^2 = 37.5%				

Positive coefficients reflect a decrease in QuickDASH equal to better function and less symptoms. The model had 17 degrees of freedom, corresponding to 16 patients per degree of freedom. Abbreviations: QuickDASH= Quick Disabilities of the arm, shoulder and hand, CI =confidence interval.

Table S7, parameter estimates for final worst-case model				
**Variable**	**Coefficient**	**95% CI**	**p-value**	**Stand. Coef.**
Baseline QuickDASH	0.62	0.48; 0.75	<0.001	0.61
Employment status				
Employed/studying	Ref.			
Unemployed/subsidised employment/ sick leave	-12.20	-18.10; -6.10	<0.001	-0.20
Pensioner	1.08	-3.11; 5.28	0.61	0.02
Professional educational level				
No education	Ref.			
Low (<2) or vocational	5.64	0.26; 11.03	<0.05	0.14
Medium (3) or high (>4)	1.65		0.58	0.04
Movement impairment classification				
Hypomobility	Ref.			
Hypermobility	-11.01	-17.06; -5.10	<0.001	-0.21
Aberrant motion	-9.03	-14.13; -3.92	<0.005	-0.22
Self-rated ability to cope with the pain	1.53	0.66; 2.41	<0.005	0.18
Health-related quality of life	11	-7; 29	0.24	0.08
Pain catastrophizing	-0.42	-0.82; -0.02	<0.05	-0.12
Intercept	-23.77	-41.10; -5.44	<0.05	-
Adjusted coefficient of determination, adjusted R^2 = 38%				

Positive coefficients reflect a decrease in QuickDASH equal to better function and less symptoms. The model had 11 degrees of freedom, corresponding to 26 patients per degree of freedom. Abbreviations: QuickDASH= Quick Disabilities of the arm, shoulder and hand, CI =confidence interval.

Supplementary analysis: backwards elimination performed from Akaike's information criterion

Table S8 Parameters for the full multivariable linear regression model (n=220)				
**Variable**	**Coefficient**	**95% CI**	**p-value**	**Stand. coef**
Baseline QuickDASH	0.62	0.41; 0.83	<0.001	0.60
Age	-0.10	-0.33; 0.13	0.40	-0.06
Sex				
Woman	Ref.			
Man	-0.39	-5.44; 4.65	0.89	-0.01
Employment status				
Employed/studying	Ref.			
Unemployed/Special terms/ sick leave	-10.89	-18.86; -2.92	<0.01	-0.17
Pensioner	1.86	-4.65; 8.38	0.57	0.04
Professional educational level				
No education	Ref.			
Low (<2) or vocational	6.33	-0.57; 13.24	0.07	0.15
Medium (3) or high (>4)	1.81	-5.58; 9.20	0.63	0.04
Movement impairment classification				
Hypomobility	Ref.			
Hypermobility	-10.10	-17.60; -2.60	<0.01	-0.19
Aberrant motion	-8.16	-14.67; -1.64	0.014	-0.20
Pain	-0.04	-1.42; 1.34	0.95	-0.005
Duration of symptoms	-0.05	-0.10; 0.01	0.09	-0.11
Self-rated ability to cope with the pain	1.20	-0.01; 2.41	0.05	0.14
Fear avoidance	-0.08	-0.68; 0.52	0.80	-0.02
Health-related quality of life	12.9	-12.6; 38.2	0.32	0.09
Mental wellbeing	0.00	-0.14; 0.14	1	0.00
Self-rated risk of persistent symptoms	0.06	-1.00; 1.11	0.91	0.01
Pain catastrophizing	-0.37	-0.95; 0.21	0.2	-0.10
Intercept	-14.28	-42.44; 13.87	0.32	-
Adjusted coefficient of determination, adjusted R^2 = 30.8%<br>Akaike's Information Criterion = 1891.16				

Positive coefficients reflect a decrease in QuickDASH equal to better function and less symptoms. The model had 17 degrees of freedom, corresponding to 13 participants per degree of freedom. Abbreviations: QuickDASH= Quick Disabilities of the arm, shoulder and hand, CI =confidence interval.

Table S9 Parameters for the final multivariable linear regression model using Akaike's Information Criterion (n=220)

**Variable**	**Coefficient**	**95% CI**	**p-value**	**Stand. Coef**
Baseline QuickDASH	0.61	0.43; 0.79	<0.001	0.59
Employment status				
Employed/studying	Ref.			
Unemployed/Special terms/ sick leave	-11.12	-18.91; -3.35	<0.005	-0.17
Pensioner	-0.22	-4.90; 5.29	0.94	-0.01
Professional educational level				
No education	Ref.			
Low (<2) or vocational	6.03	-0.38; 12.76	0.07	0.15
Medium (3) or high (>4)	0.94	-5.18; 8.97	0.60	0.04
Movement impairment classification				
Hypomobility	Ref.			
Hypermobility	-10.36	-17.04; -2.56	<0.005	-0.18
Aberrant motion	-8.57	-13.80; -1.48	<0.005	-0.18
Duration of symptoms	-0.04	-0.09; 0.01	0.08	-0.10
Self-rated ability to cope with the pain	1.16	0.02; 2.30	<0.05	0.13
Health-related quality of life	12.8	-10.7; 36.2	0.29	0.09
Pain catastrophizing	-0.38	-0.89; 0.13	0.15	-0.10
Intercept	-20.07	-43.45; 3.30	0.09	-
Adjusted coefficient of determination, adjusted R^2 = 32.5%<br>Akaike's information criterion = 1880.12				

Positive coefficients reflect a decrease in QuickDASH equal to better function and less symptoms. The model had 10 degrees of freedom, corresponding to 23 participants per degree of freedom. Abbreviations: QuickDASH= Quick Disabilities of the arm, shoulder and hand, CI =confidence interval.
